# The effect of enhanced recovery after minimally invasive esophagectomy: a randomized controlled trial

**DOI:** 10.1007/s00464-022-09385-6

**Published:** 2022-06-30

**Authors:** Yaxing Shen, Xiaosang Chen, Junyi Hou, Youwen Chen, Yong Fang, Zhanggang Xue, Xavier Benoit D’Journo, Robert J. Cerfolio, Hiran C. Fernando, Alfonso Fiorelli, Alessandro Brunelli, Jing Cang, Lijie Tan, Hao Wang, Written on behalf of the AME Thoracic Surgery Collaborative Group

**Affiliations:** 1 Department of Thoracic Surgery, Zhongshan Hospital, Fudan University, 180 Fenglin Road, Shanghai, China; 2 Department of Thoracic Surgery, National Cancer Center/National Clinical Research Center for Cancer/Cancer Hospital, Chinese Academy of Medical Sciences and Peking Union Medical College, Beijing, 10021 China; 3 Department of Anesthesiology, Zhongshan Hospital, Fudan University, 180 Fenglin Road, Shanghai, China; 4 Department of Thoracic Surgery and Diseases of Esophagus, Aix-Marseille University, North Hospital, Chemin des Bourrely, 13915 Marseille Cedex 20, France; 5 Department of Cardiothoracic Surgery, New York University Langone Health, New York, NY USA; 6 Department of Cardiothoracic Surgery, Allegheny General Hospital, Pittsburgh, PA USA; 7 Thoracic Surgery Unit, Università Della Campania Luigi Vanvitelli, Naples, Italy; 8 Department of Thoracic Surgery, St. James’s University Hospital, Bexley Wing, Beckett Street, Leeds, LS9 7TF UK

**Keywords:** Minimally invasive esophagectomy, MIE, Enhanced recovery after surgery, ERAS, Morbidity, Length of stay, LOS

## Abstract

**Background:**

The purpose of this randomized controlled trial was to determine if enhanced recovery after surgery (ERAS) would improve outcomes for three-stage minimally invasive esophagectomy (MIE).

**Methods:**

Patients with esophageal cancer undergoing MIE between March 2016 and August 2018 were consecutively enrolled, and were randomly divided into 2 groups: ERAS+group that received a guideline-based ERAS protocol, and ERAS- group that received standard care. The primary endpoint was morbidity after MIE. The secondary endpoints were the length of stay (LOS) and time to ambulation after the surgery. The perioperative results including the Surgical Apgar Score (SAS) and Visualized Analgesia Score (VAS) were also collected and compared.

**Results:**

A total of 60 patients in the ERAS+ group and 58 patients in the ERAS- group were included. Postoperatively, lower morbidity and pulmonary complication rate were recorded in the ERAS+ group (33.3% vs. 51.7%; *p* = 0.04, 16.7% vs. 32.8%; *p* = 0.04), while the incidence of anastomotic leakage remained comparable (11.7% vs. 15.5%; *p* = 0.54). There was an earlier ambulation (3 [2–3] days vs. 3 [3–4] days, *p* = 0.001), but comparable LOS (10 [9–11.25] days vs. 10 [9–13] days; *p* = 0.165) recorded in ERAS+ group. The ERAS protocol led to close scores in both SAS (7.80 ± 1.03 vs. 8.07 ± 0.89, *p* = 0.21) and VAS (1.74 ± 0.85 vs. 1.78 ± 1.06, *p* = 0.84).

**Conclusions:**

Implementation of an ERAS protocol for patients undergoing MIE resulted in earlier ambulation and lower pulmonary complications, without a change in anastomotic leakage or length of hospital stay. Further studies on minimizing leakage should be addressed in ERAS for MIE.

Surgical resection of esophageal cancer offers the chance for cure, yet carries a high risk of morbidity and mortality [1]. Currently, the reported incidence of complications remains high even with the implementation of minimally invasive techniques. Morbidity after esophagectomy can result in prolonged length of hospital stay and recovery [2, 3]. Although enhanced recovery after surgery (ERAS) protocols have been established in other surgical disciplines [4, 5], its role in patients undergoing esophagectomy remains less studied [6].

In the year 2014, a systemic review of ERAS for esophagectomy included only 6 retrospective studies [7]. The volume of literature regarding this topic increased almost fivefold in the following 5 years, leading to a publication of guidelines for esophagectomy by the ERAS society [8], which covered perioperative care for patients underwent esophagectomy [9–11]. To date, although various ERAS protocols have been used across different studies, a shortened length of hospital stay (LOS) was consistently reported regardless of the protocol used [12]. As such, uncertainty of what significance a certain protocol plays toward a successful outcome was raised. Additionally, it is unclear how ERAS further improve outcomes with respect to minimally invasive esophagectomy (MIE), which has been clearly shown to enhance recovery [13, 14].

As a high-volume center focused on thoracoscopic esophagectomy [15], our previous study was referenced in the ERAS societal Guidelines [16]. Based on our accumulative experience from MIE, we herein proposed our ERAS protocol in this randomized trial and clarify its role in esophagectomy.

## Methods

### Study design

This was a prospective, randomized controlled trial conducted at the Department of Thoracic Surgery, Zhongshan Hospital, Fudan University from March 2016 to August 2018. The study design planned according to CONSORT (Consolidated Standards of Reporting Trials) guidelines was approved by the Ethics Committee of Zhongshan Hospital, Fudan University (B2016-043R), and the trial was registered at Chinese Clinical Trial Registry (ChiCTR-IOR-17010631). All enrolled patients received detailed information of the procedures and goals of the study. They were informed that their participation was voluntary, and that they could withdraw their consent to participate at any time during the study without any consequences for their care. All patients provided written informed consent before entering the study.

### Participants

Patients with esophageal cancer who were eligible for 3-stage MIE were consecutively enrolled in the trial. Tumors were clinically staged by endoscopy, tissue biopsy, thoraco-abdominal computed tomography (CT), and positron emission tomography (PET)-CT scan. Based on the clinical finding, eligibility for MIE and inclusion in the study were (1) Clinical stage T_1-3_N_0_M_0_ /ypT_1-3_N_0_M_0_; (2) American Society of Anesthesiologists (ASA) score of I-II; (3) No previous cervical or thoracic surgery; (4) No history of prior malignancy.

The exclusion criteria for MIE and for the study were as follows: (1) Severe preoperative comorbidities; (2) Forced expiratory volume in one second (FEV_1_) < 50%, or ejection fraction (EF) < 50% as predicted, or organ failure; (3) Receiving preoperative corticosteroid treatment; (4) The presence of a cognitive disorder.

Patients were also excluded from the study if (1) Tumor invasion to adjacent structures was identified intraoperatively; (2) The procedure was converted to an open operation or salvage esophagectomy; (3) There were significant intraoperative complications; (4) The patient refused to participate or continue during any period of the study.

### Randomization

Enrolled patients were randomly assigned to receive MIE with an enhanced recovery after surgery protocol (ERAS +), or MIE without ERAS (ERAS-). Randomization was performed by an independent registered nurse using sequentially numbered, sealed envelopes containing information that disclosed the group to which the patient was assigned.

### Anesthesia and analgesia

MIE was performed with general anesthesia and single lumen intubation, combined with thoracic epidural analgesia. For postoperative pain management, epidural analgesia was administered in the form of 15 mL 0.1% bupivacaine injected into the epidural space before the surgery ended. Postoperatively, patient-controlled epidural analgesia (PCEA) was administered using an electronic pump. PCEA consisted of bupivacaine (262.5 mg), fentanyl (0.5 mg), and morphine (5 mg), which were diluted in normal saline to a final volume of 250 mL. The analgesia pump settings were load volume of 4 mL, background dose of 3 mL/h, bolus dose of 2 mL, and lockout time of 10 min. When the standard analgesia was unsatisfactory, the background dose was increased to 6 mL/h after the level of analgesia and stable blood pressure were confirmed. Intravenous oxycodone (5 mg) was used as rescue analgesia.

### Surgery

All operations were performed by the same senior surgeon and consisted of 3 stages. (1) The thoracic stage included esophageal mobilization and mediastinal lymphadenectomy under 8 mm Hg artificial CO_2_ pneumothorax [17], with preservation of the thoracic duct [18]. (2) The abdominal stage included laparoscopic gastric mobilization under 12 mm Hg artificial CO_2_ pneumoperitoneum. (3) Gastric conduit formation was carried out through mini-laparotomy without pyloroplasty [19]. The gastric conduit was pulled up to the cervical incision through the posterior mediastinal route, and a circular stapler was used to accomplish the gastroesophageal anastomosis. A mediastinal drainage tube was placed through the cervical incision. The operation concluded with closure of the cervical and abdominal incisions.

The Surgical Apgar Score (volume of estimated blood loss, the lowest intraoperative mean arterial pressure, and the lowest intraoperative heart rate) was recorded for all patients to assess surgical stress [20].

### Interventions

The ERAS protocol used was based on the previously published guidelines [8]. Some of the ERAS protocol steps had already been introduced as conventional care at our medical center (i.e., preoperative multidisciplinary team board discussion [21], use of MIE [15], avoidance of pyloroplasty/abdominal drainage [19]).

The differences of the ERAS+ and ERAS- pathways are outlined in Table 1, and the components of ERAS+ and ERAS- are summarized below.

**Table 1:** Differences between ERAS+ and ERAS − groups

	ERAS+	ERAS −
Preoperative pathways		
Rehabilitation training	+	−
Fasting strategy		
Solids	6 h	10 h
Fluids	2 h	4 h
Carbohydrate loading supplement	+	−
Intraoperative pathways		
Goal directed fluid therapy	+	−
Drainage placement		
Nasogastric tube	−	+
Nasojejunal tube	+	+
Chest tube	+	+
Mediastinal tube	+	+
Postoperative pathways		
ICU stay	−	POD 1
Early mobilization	+	−
Removal of chest drain (drainage volume)	< 200 mL/d	< 100 mL/d
Oral intake	POD 5	POD 7

*ERAS* enhanced recovery after surgery, *ICU* intensive care unit, *POD* postoperative day

## ERAS+ group protocols

### Preoperative ERAS+ training

#### Rehabilitation training

Patients in ERAS+ group were educated preoperatively by a trained nurse on (1) diaphragmatic breathing (2) effective coughing, and (3) aerobic ambulation. Patients were trained until he or she could accurately repeat each element of the rehabilitation training. Based on the education, patients were requested to perform self-directed training until the operation day, and then resume training 1 day after surgery until discharge.

#### Modified fasting strategy

ERAS+ patients were instructed to fast 6 h for solid foods and 2 h for clear fluids before surgery (instead of 10 h for solids and 4 h for clear fluids for ERAS− patients). At 2–6 h before surgery, a 10% glucose fluid (8–10 mL/kg) was administered to ERAS+  patients orally.

### Intraoperative for ERAS+ protocol

#### Fluid strategy

An intraoperative goal-directed fluid therapy pathway was used for ERAS+ patients. Patients received a continuous intraoperative infusion of balanced solution at a fixed amount of 2 mL/kg (ideal body weight)/hour, and hemodynamic management was achieved by monitoring cardiac index (CI) and stroke volume variation (SVV). A LiDCO rapid system (LiDCO, London, United Kingdom) was used to monitor the real-time hemodynamic status and facilitate stroke volume optimization. If hypotension occurred with a CI < 2.4 and SVV ≥ 12% during the operation, 200 mL colloid solution was administered. If the stroke volume increased > 10% after colloid loading, an extra 200 mL colloid solution was infused. If the SVV was < 12%, norepinephrine was administered.

#### Tube management

During the procedure, a nasojejunal tube, chest pleural drain, and mediastinal drain were placed. Cervical or abdominal drainage was not used in ERAS+  cases. At the end of the surgery, immediate extubation was performed and patients were transferred to the ward. The nasojejunal tube was left in place for postoperative nutritional support. The chest tube was removed when drainage was < 200 mL/d, and there were no signs of pneumothorax.

### Postoperative pathways for ERAS+ protocol

#### Nutritional support

Patients were fed with enteral nutrition through the nasojejunal tube from postoperative day (POD) 2 to POD 4. After anastomotic leakage was ruled out by a contrast swallow test on POD 5, patients were begun on oral intake. If there was no sign of fever or discomfort after beginning oral intake, the remaining tubes were removed and discharge was planned.

#### Physical therapy

Physical and respiratory rehabilitation started on POD 1, with progressive mobilization (sitting in a bedside chair on POD 1–2, assisted walking on POD 3–4, and then daily increases in frequency until the patient was able to walk independently). The results of physical therapy were recorded by a registered nurse.

### ERAS− group protocols

The ERAS- patients were asked to fast 10 h for solid foods and 4 h for clear fluids before surgery. The patients received a conventional intraoperative infusion of a balanced solution, and fluid management was adjusted primarily based on the “4–2-1 rule” for fluid loss. If arterial blood pressure decreased by > 30%, the speed of fluid administration was increased, and norepinephrine was used to maintain the mean arterial pressure (MAP) ≥ 65 mm Hg. A nasogastric tube, nasojejunal tube, chest pleural drain, and mediastinal drain were routinely placed during the procedure. The patients were transferred to the ICU and remained there until POD 1. No specific mobilization targets were set for this group.

The nasogastric tube was removed on POD 1 unless excessive bleeding was noted. The chest drainage tube was removed when drainage was < 100 mL/d, and there were no signs of pneumothorax. Patients received enteral nutrition through the nasojejunal tube from POD 2 to POD 6. After anastomotic leakage was ruled out by a contrast swallow test on POD 7, patients resumed an oral intake. If there was no sign of fever or discomfort after beginning oral intake, the remaining tubes were removed and discharge was planned.

### Primary study endpoint

A comparison of postoperative morbidity between the ERAS+ and ERAS- groups was the primary endpoint of the study. Morbidities were classified based on the standard list proposed by the Esophageal Complications Consensus Group [22]. Chest X-ray and routine blood testing were conducted to identify possible pulmonary complications after surgery. Cardiac complications were recorded as cardiac arrest requiring cardiopulmonary resuscitation or dysrhythmia requiring extra medical treatment. Gastrointestinal complications were anastomotic leakage or conduit failure confirmed according to the radiologic evidence from swallow test.

### Secondary study endpoints

The length of hospital stay and the time to ambulation after the surgery of the 2 groups were the secondary endpoints of the study. The length of hospital stay was recorded from the date of the surgery until the date of discharge. Discharge was planned when patient reached free morbidity status at (1) vital signs were stable, (2) all drainage tubes were removed, and (3) no interventions were required due to complications. The time to ambulation was recorded by the registered nurse caring for the patient under the guidance of the research agents.

### Sample size

The sample size calculation was based on the primary endpoint. A comprehensive review of MIE-associated complications in high-volume centers demonstrated an overall complication rate of 50% [23, 24]. We calculated that each group required 55 patients for a difference to be detected in an overall complication rate of 25% in the ERAS+group versus 50% in the ERAS- group (2-sided test; alpha level, 0.05; beta level, 0.80).

### Statistical analysis

Data were summarized as the mean and standard deviation or median and interquartile range, as appropriate, for continuous variables; and absolute number and percentage for categorical variables. Continuous variables were compared by Student’s *t* test or Mann–Whitney U test, as appropriate, and categorical variables were compared by chi-square test or Fisher’s exact test, as appropriate. A value of *P* < 0.05 was considered to indicate statistical significance. Statistical analysis was performed using GraphPad Prism 6.0 (GraphPad Software, CA).

## Results

### Patient characteristics

During the study period, 134 consecutive patients were found to be eligible for the study. Of the 134 patients, 12 patients were excluded due to interstitial lung disease (*n* = 3), unresectable tumors (*n* = 6), and refusal to participate (*n* = 3). Thus, a total of 122 patients were included in the study and randomized to the ERAS+ group (*n* = 62) and the ERAS- group (*n* = 60). Subsequently, 4 patients were excluded from the study and analysis: 1 patient in the ERAS+ group experienced major intraoperative bleeding from a variant pulmonary vein close to the subcarinal lymph node, 1 patient in the ERAS− group was converted to open thoracotomy due to tumor invasion to the left main bronchus identified intraoperatively; 2 patients (1 in the ERAS+ and 1 in the ERAS− group) experienced iatrogenic injury to the right gastroepiploic artery of the gastric conduit. These 4 patients were treated based on their complications.

**Fig. 1:**
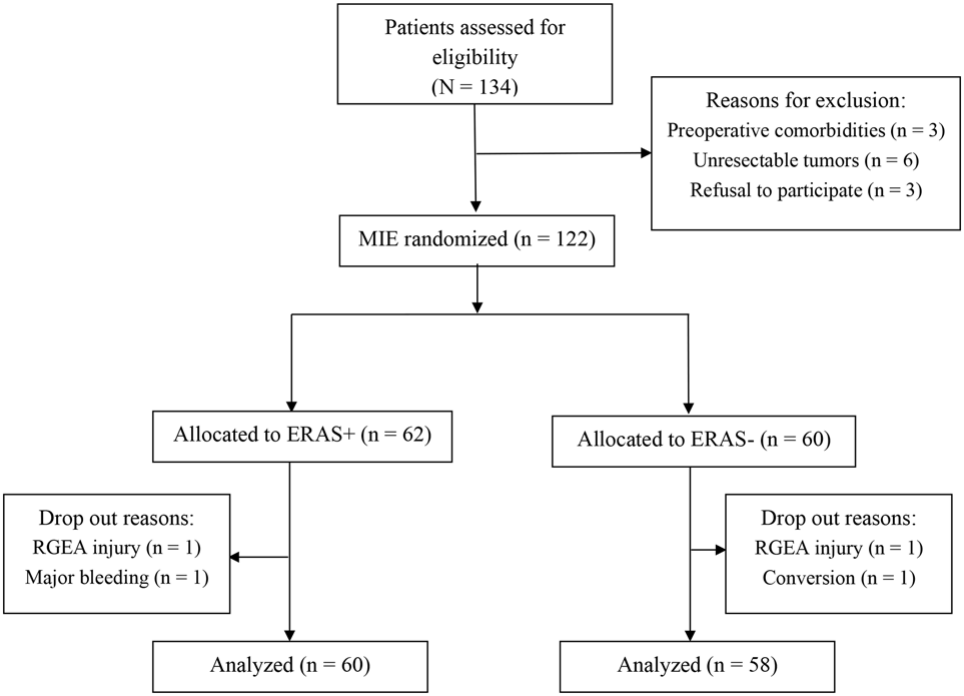
Flowchart of patient inclusion, allocation, and analysis. CI, cerebral infraction; ERAS, enhanced recovery after surgery; MIE, minimally invasive esophagectomy; RGEA, right gastroepiploic artery

A flow diagram of patient inclusion is shown in Fig. 1. The 2 study groups were well balanced without significant differences with respect to demographic characteristics and clinical characteristics such as ASA class, tumor stage, and neoadjuvant therapy. Patient baseline data are summarized in Table 2.

**Table 2:** Patient baseline characteristics

	ERAS+group (*n* = 60)	ERAS- group (*n* = 58)	*p* value
Age, years	62.28 ± 6.74	62.33 ± 7.47	0.97†
BMI, kg/m^2^	23.58 ± 2.93	23.24 ± 2.92	0.53†
Albumin, g/L	42.87 ± 6.24	42.88 ± 6.70	0.99†
Sex			
Male	44 (73.3)	48 (82.8)	0.22*
Female	16 (26.7)	10 (17.2)	
ASA score			
I	38 (63.3)	43 (74.1)	0.21*
II	22 (36.7)	15 (25.9)	
Pathology			
SCC	58 (96.7)	57 (98.3)	0.98**
NC	2 (3.3)	1 (1.7)	
T stage			
T_0_	2 (3.3)	1 (1.7)	0.12**
T_1_	21 (35.0)	14 (24.1)	
T_2_	19 (31.7)	18 (31.0)	
T_3_	18 (30.0)	25 (43.1)	
Neoadjuvant treatment			
None	48 (80.0)	49 (84.5)	0.74*
Chemoradiotherapy	12 (20.0)	9 (15.5)	

Data reported as mean ± standard deviation, or count

*ASA* American Society of Anesthesiologists, *BMI* body mass index, *ERAS* enhanced recovery after surgery, *NC* neuroendocrine carcinoma, *SCC* squamous cell carcinoma

^†^Mann–Whitney test

*Chi-square test

**Fisher’s exact test

### Primary endpoint

The overall complication was 42.3% from this cohort, with pulmonary (24.6%) and gastrointestinal (14.4%) complications listed as the most common morbidities. The complication rate was lower in ERAS+ group compared to ERAS− group (20 of 60 [33.3%] vs 30 of 58 [51.7%]; *p* = 0.04). In terms of pulmonary complications, the incidence from the ERAS+ group was significantly lower than the ERAS- group (10 of 60 [16.7%] vs 19 of 58 [32.8%]; *p* = 0.04). Three patients developed cardiac complications that required treatment for arrhythmia (2 of 60 [4.0%] in the ERAS+ group and 1 of 58 [1.7%] in the ERAS- group; *p* = 0.98). There was no significant difference in the incidence of gastrointestinal complications between the 2 groups (7 of 60 [11.7%] in the ERAS+ group vs 10 of 58 [20.7%] in the ERAS- group; *p* = 0.39). One patient in the ERAS+ group developed a wound infection. Outcomes are summarized in Table 3.

**Table 3:** Postoperative morbidity and mortality

Morbidity and mortality	Patients, No. (%)		Between-group difference, RD (95% CI)	*p* value
	ERAS+group (*n* = 60)	ERAS- group (*n* = 58)		
Morbidity	20 (33.3)	30 (51.7)	18.4 (0.6 to 36.2)	0.04*
Pulmonary	10 (16.7)	19 (32.8)	16.7 (0.5 to 31.6)	0.04*
Pneumonia	4 (6.7)	3 (5.2)		1.00**
Atelectasis	2 (3.3)	9 (15.5)		0.03**
Pleural effusion	2 (3.3)	3 (5.2)		0.97**
ALI/ARDS	2 (3.3)	4 (6.9)		0.64**
Cardiac	2 (3.3)	1 (1.7)		0.98**
Gastrointestinal	7 (11.7)	10 (17.2)	5.6 (− 7.1. to. 18.3)	0.39*
Anastomotic leakage	7 (11.7)	9 (15.5)	3.9 (− 8.5 to 16.2)	0.54*
Delayed conduit emptying	0	1 (1.7)		0.99**
Wound	1 (1.7)	0		0.99**
Mortality	1 (1.7)	0 (0)		0.98*

*ALI* acute lung injury, *ARDS* acute respiratory distress syndrome, *ERAS* enhanced recovery after surgery

*Chi-square test

**Fisher’s exact test

### Secondary endpoints

Patients in the ERAS+ group began ambulation earlier than patients in the ERAS- group (3 [2, 3] days vs. 3 [3, 4] days; *p* = 0.0013). However, the total length of hospital stay was similar between the 2 groups (10 [9–11.25] days in the ERAS+ group vs. 10 [9–13] days in the ERAS- group; *p* = 0.165) (Table 4).

**Table 4:** Time to ambulation and length of hospital stay

	ERAS+group (*n* = 60) Median (IQR)	ERAS- group (*n* = 58) Median (IQR)	*p* value
Time to ambulation, days	3 (2–3)	3 (3–4)	0.001†
Length of hospital stay, days	10 (9–11.25)	10 (9–13)	0.165†

Data reported as mean ± standard deviation, or count (percentage)

^†^Mann–Whitney test

In ERAS+ group, 1 patient was readmitted on POD 14 for dyspnea and fever, and died on the same day because of cardiac arrest. The patient was recorded as a case of postoperative mortality; however, there was no significant difference in 30-day mortality between the 2 groups (1.7% in the ERAS+ group vs 0% in the ERAS- group; *p* = 0.99) (Table 3).

### Peri-operative findings

Intraoperatively, patients in the ERAS+ group received less crystalloid fluid (555.9 ± 165.9 mL vs. 2498.2 ± 582.8 mL; *p* < 0.001), and more colloid fluid (361.0 ± 143.9 mL vs. 0 ± 0 m; *p* < 0.001). The volume of total intraoperative fluid administered was significantly lower in the ERAS+ group (916.9 ± 229.9 mL vs. 2498.2 ± 582.8 mL; *p* < 0.001). There was no significant difference in SAS between the groups (7.8 ± 1.0 vs. 8.1 ± 0.9; *p* = 0.21). Postoperatively, the visual analog scale (VAS) pain score was comparable between the 2 groups (1.7 ± 0.9 vs. 1. 8 ± 1.1; *p* = 0.84). The results are summarized in Table 5.

**Table 5:** Peri-operative data

Factor	ERAS+group (*n* = 60)	ERAS- group (*n* = 58)	*p* value
Total infusion volume, mL	916.9 ± 229.9	2498.2 ± 582.8	< 0.0001†
Crystalloid infusion, mL	555.9 ± 165.9	2498.2 ± 582.8	< 0.0001†
Colloid infusion, mL	361.0 ± 143.9	0 ± 0	< 0.0001†
SAS, mean (SD)	7.8 ± 1.0	8.1 ± 0.9	0.21†
VAS, mean (SD)	1.7 ± 0.9	1.8 ± 1.1	0.84†

*ERAS* enhanced recovery after surgery, *SAS* Surgical Apgar Score, *VAS* Visualized Analgesia Score

^†^Mann–Whitney test

## Discussion

This prospective randomized controlled trial assessed the outcomes of the ERAS protocol in patients receiving MIE at our high-volume center. Our results showed lower complication rates, especially with respect to pulmonary complications, in patients undergoing MIE who received ERAS. The ERAS+ group achieved earlier ambulation, while there was no significant difference in gastrointestinal leakage or overall length of hospital stay between the 2 groups.

There are many reasons for a delayed recovery after surgical resection of esophageal cancer. In a recent study, Parise et al. [25] reported that prolonged stay after esophagectomy was associated with both the patient clinical characteristics (ASA score > 3, failure of postoperative mobilization) and factors related to the operation (surgery duration > 255 min and “non-hybrid esophagectomy”). Therefore, an optimal ERAS protocol should include a wide spectrum of perioperative elements. Based on the ERAS society’s guideline for esophageal cancer, many of the recommended pathways have been implemented as the standard care for patients at our medical center [8, 26], and accumulated experience from our previous studies [15, 16] led to the proposal of an ERAS study to evaluate its effectiveness on patients undergoing MIE.

In terms of the protocol design, the patients’ compliance is an important consideration since deviations from ERAS protocol can impact outcomes [27]. In this study, the high adherence rate can be explained by the simple structure of the study protocol. From the patients’ perspective, the ERAS protocol only included the rehabilitation training, the modified fasting strategy, and the early ambulation. Notably, the instructions for these components were easier to follow when compared to studies with more complex pathways [28]. Also, we excluded 2 patients who developed iatrogenic injury to the conduit blood supply because an early failure in gastroesophageal reconstruction would interfere with oral intake on POD 5–7, which is one of the ERAS elements.

ERAS studies performed in other specialties usually focused on decreasing the postoperative length of hospital stay [5, 29]. However, since McKeown esophagectomy involves multiple operation fields and distal isotopic anastomosis [30], we gave higher priority to morbidity than LOS since the complications remain high even in MIE, and are considered the leading contributor to the prolonged hospital stay [13, 24]. Given that LOS could be subjective and varied based on institutional preferences [31, 32], and the morbidity-free status was also included in the discharge criteria in our study, the incidence of morbidity was therefore used as the primary end point of the study.

To minimize complications following MIE, a series of studies focused on the role of GDT as intraoperative hypovolemia can lead to inadequate tissue oxygen delivery and contribute to postoperative complications [33, 34]. On the other hand, hypervolemia can result in tissue edema and delay the functional recovery of the gastrointestinal tract [35]. Hence, the GDT pathway was centralized in our ERAS protocol, aiming at enhanced recovery under individualized intraoperative infusion. In this study, we observed that ERAS+ group experienced significant lower volume of intravenous administration than ERAS− group, since the colloid infusion has a larger volume expansion effect and longer maintenance period in compared to crystalloid infusion. Hence, the colloid infusion in ERAS+ group costs lower volume to achieve comparable hemodynamic endpoints in compared to crystalloids [36], resulting in less interstitial fluid overload and accelerate the functional recovery following MIE.

In addition to the GDT pathway, the modified fasting strategy also contributed to a lower infusion volume in the ERAS+ group. In this pathway, preoperative oral intake (10% glucose fluid before the surgery) was supplied for physiological requirement, which resulted in less volume demand during the operation. Due to the combination of a modified fasting strategy and the GDT pathways, hemodynamic stability was achieved with the administration of a lower volume of intraoperative intravenous fluids. Similar findings were also reported in a recent study of colorectal cancer surgery, which concluded that a high volume of infusion on the day of surgery would increase cardiopulmonary complications [37].

In our study, hemodynamic stability was evaluated using the SAS in order to identify potential inadequate perfusion [38]. In a report from Nakagawa et al. [39], the author reported that a SAS < 5 indicated hemodynamic instability and was a significant risk factor for morbidity. In our study, the average SAS score of both groups was over 5, which indicated the absence of hemodynamic compromise. This was further corroborated by the comparable incidence of anastomotic leakage postoperatively, an event which has been associated with perfusion of the gastric conduit intraoperatively [40].

In this study, the ERAS protocol showed preference in minimizing pulmonary complications, yet the incidence of anastomotic leakage was not affected, and even an earlier ambulation in ERAS+ group did not translate into a shorter length of hospital stay. The anastomotic leakage, which usually presented around POD 7 when patients have followed most of the ERAS pathways, is another important reason for delayed discharge besides pulmonary complications. To further decrease the LOS in esophagectomy, early identification and optimal intervention of anastomotic leakage could be considered in the future ERAS protocols. Yu et al. [41] reported that an elevated amylase level in cervical drainage on POD 3 was a predictive of anastomotic leakage after esophagectomy. In these cases, awareness and early detection may help avoid the need for contrast radiological studies, and accelerate hospital discharge through earlier intervention [42].

Our study has several limitations that need to be addressed before drawing definitive conclusions. Firstly, due to the study design, the hospital length of stay could be subjective and influenced by an individual surgeon’s perspective. Secondly, we did not examine how each pathway contributed to acceleration of recovery; therefore, a standardized ERAS pathway in esophagectomy would be required in future studies [43]. Thirdly, this study did not evaluate the long-term effect of ERAS on esophageal cancer patients.

## Conclusions

Implementation of an ERAS protocol for patients undergoing MIE resulted in earlier ambulation and a lower incidence of pulmonary complications, without a change in overall length of hospital stay. Further studies based on larger populations are required to confirm our findings.
